# Preclinical Studies of Immunogenity, Protectivity, and Safety of the Combined Vector Vaccine for Prevention of the Middle East Respiratory Syndrome

**DOI:** 10.32607/actanaturae.11042

**Published:** 2020

**Authors:** I. V. Dolzhikova, D. M. Grousova, O. V. Zubkova, A. I. Tukhvatulin, A. V. Kovyrshina, N. L. Lubenets, T. A. Ozharovskaia, O. Popova, I. B. Esmagambetov, D. V. Shcheblyakov, I. M. Evgrafova, A. A. Nedorubov, I. V. Gordeichuk, S. A. Gulyaev, A. G. Botikov, L. V. Panina, D. V. Mishin, S. Y. Loginova, S. V. Borisevich, P. G. Deryabin, B. S. Naroditsky, D. Y. Logunov, A. L. Gintsburg

**Affiliations:** N.F. Gamaleya National Research Center for Epidemiology and Microbiology of the Ministry of Health of the Russian Federation, Moscow, 123098 Russia; I.M. Sechenov Institute of Evolutionary Physiology and Biochemistry of the Russian Academy of Sciences, Moscow, 119435 Russia; M.P. Chumakov Federal Scientific Center for Research and Development of Immune-and-Biological Products of the Russian Academy of Sciences, Moscow, 108819 Russia; The 48th Central Research Institute of the Ministry of Defense of the Russian Federation, Moscow, 141306 Russia

**Keywords:** adenoviral vector, Middle East Respiratory Syndrome (MERS), immunogenicity, safety assessment

## Abstract

The Middle East Respiratory Syndrome (MERS) is an acute inflammatory disease of the respiratory system caused by the MERS-CoV coronavirus. The mortality rate for MERS is about 34.5%. Due to its high mortality rate, the lack of therapeutic and prophylactic agents, and the continuing threat of the spread of MERS beyond its current confines, developing a vaccine is a pressing task, because vaccination would help limit the spread of MERS and reduce its death toll. We have developed a combined vector vaccine for the prevention of MERS based on recombinant human adenovirus serotypes 26 and 5. Studies of its immunogenicity have shown that vaccination of animals (mice and primates) induces a robust humoral immune response that lasts for at least six months. Studies of the cellular immune response in mice after vaccination showed the emergence of a specific CD4^+^ and CD8^+^ T cell response. A study of the vaccine protectivity conducted in a model of transgenic mice carrying the human DPP4 receptor gene showed that our vaccination protected 100% of the animals from the lethal infection caused by the MERS-CoV virus (MERS-CoV EMC/2012, 100LD_50_ per mouse). Studies of the safety and tolerability of the developed vaccine in rodents, rabbits, and primates showed a good safety profile and tolerance in animals; they revealed no contraindications for clinical testing.

## INTRODUCTION

The Middle East Respiratory Syndrome (MERS) is an acute inflammatory disease of the respiratory system that was first diagnosed in June 2012 in Saudi Arabia [1, 2]. The disease is caused by the MERS-CoV coronavirus, a member of the genus *Betacoronavirus *of the family *Coronaviridae*. One-humped camels are the natural reservoir of the virus; human infection occurs through contact with camels and consumption of unpasteurized camel milk; an aerosol transmission of infection is also possible [3, 4]. According to the WHO, a total of 2,458 laboratory-confirmed cases of MERS had been registered by September 12, 2019, 848 of which resulted in a fatal outcome (a 34.5% mortality rate) [5]. Most MERS cases were registered in Saudi Arabia [6]. However, the disease was also detected in 27 other countries (the United Arab Emirates, South Korea, Yemen, etc.); cases of imported infection were reported in Europe, North Africa, and North America [5]. Because of the lack of effective preventive and therapeutic drugs for MERS, the high mortality rate of the disease, and the widespread character of the infection reservoir, WHO experts classify MERS-CoV as a virus with the potential to cause a pandemic. There have been no cases of MERS in Russia. However, due to the high mortality of MERS and the continuing threat that it could spread outside the endemic areas [5], development of a vaccine is an urgency. Vaccination can limit the spread of MERS and reduce its mortality [7].

To date, several candidate vaccine preparations based on a protective antigen, MERS-CoV S glycoprotein and its derivatives (S1 subunit, receptor-binding domain), are known: vector vaccines (based on recombinant adenoviruses and vaccinia virus), a DNA vaccine based on plasmid DNA, as well as vaccines based on recombinant proteins and virus-like particles [8-15]. Since the formation of a humoral and cellular immune response is important to protect against MERS-CoV, the use of recombinant viral vectors for antigen delivery seems promising for the development of anti- MERS vaccines. These vectors provide long-term expression of the antigen in the cells of the immunized organism, which results in a protective immune response as early as after the first or second immunization. Repeated vaccination is effective in inducing the most pronounced and lasting immune response, while heterologous vaccination involving the use of different viral vectors for primary and secondary immunization is the most optimal regimen. This regimen was successfully implemented in the development of a vaccine against the disease caused by the Ebola virus; the vaccine has been registered in the Russian Federation for medical use and already undergone post-registration clinical trials in the African Republic of Guinea [16].

We have developed a combined vector vaccine for the prevention of MERS based on recombinant human adenovirus serotypes 26 and 5 expressing MERS-CoV glycoprotein (MERS-CoV EMC/2012 isolate). Here, we present the results of a study of the post-vaccination humoral and cellular immune responses in mice and primates, as well as the results of preclinical studies of the safety of the developed vaccine against MERS.

## EXPERIMENTAL

**Study drug**

The combined vector vaccine against MERS consists of two components.

Component 1 presents viral particles of recombinant human adenovirus serotype 26 carrying the gene for the receptor-binding domain of MERS-CoV glycoprotein, 10^11^ viral particles (v.p.) per dose.

Component 2 presents viral particles of recombinant human adenovirus serotype 5 carrying the gene for the full-length MERS-CoV glycoprotein and the gene for the receptor-binding domain of MERS-CoV glycoprotein, 10^11^ viral particles (v.p.) per dose.

Both components are lyophilisates for the preparation of solutions for intramuscular administration. The drug was obtained in compliance with the conditions of biotechnological production at the Medgamal branch of the Gamaleya National Research Center for Epidemiology and Microbiology of the Ministry of Health of the Russian Federation.

**Laboratory animals**

All experiments on animals were carried out in strict accordance with the recommendations of the National Standard of the Russian Federation (GOST R 53434- 2009, “Principles of Good Laboratory Practice”). Six-week-old female C57BL/6 mice (18–20 g) were purchased from the Pushchino Breeding Facility (Russia). Transgenic F1 hybrid mice were obtained by crossing transgenic homozygous +/+ males carrying the human DPP4 receptor gene (hDPP4) (Medical University of Texas, USA) and non-transgenic C57BL/6 females (Pushchino, Russia). Expression of the transgene in F1 hybrid mice was confirmed by immunoblotting. All mice had free access to water and food and were housed in an ISOcage animal housing system (Tecniplast, Italy).

Common marmosets (*Callithrix jacchus*) were born and kept in a specialized animal facility at the Chumakov Federal Scientific Center for Research and Development of Immune-and-Biological Products RAS (Moscow, Russia). The animals were kept at the Laboratory for Modeling Immunobiological Processes with the Experimental Clinic of Callitrichidae (Chumakov Federal Scientific Center for Research and Development of Immune-and-Biological Products RAS) in accordance with the requirements for housing laboratory primates. All experimental procedures with marmosets were carried out by a specialist who had received certification from the Federation of European Laboratory Animal Science Associations (FELASA) and completed a course on working with primates (“Laboratory Animal Science for Researchers: Non-Human Primates,” Karolinska Institute, Stockholm, Sweden). All animals were identified by a radio chip implanted subcutaneously and having a unique 15-digit code (Globalvet, Moscow).

**Immunization of mice and marmosets and collection of their serum samples**

The mice were immunized intramuscularly using the widest possible dose range, 5×10^11^ to 105 v.p. per mouse. Immunization was carried out twice successively with component 1 and then component 2 with a 21-day interval. Mouse serum samples were collected at the following time points: 14 and 28 days, three and six months after immunization.

The marmosets were immunized intramuscularly at a dose of 1011 v.p. per animal. Immunization was conducted twice successively with component 1 and then component 2 with a 21-day interval. Plasma samples were collected at the following time points: before immunization, seven and 24 days, as well as three and six months, after immunization.

**Determination of antibody titer by enzyme-linked immunosorbent assay (ELISA)**

The titer of glycoprotein-specific antibodies in serum/ plasma was determined by enzyme immunoassay. The following recombinant proteins were used: S glycoprotein (40069-V08B; Sino Biological, China) and RBD (40071-V08B1; Sino Biological). A PBS solution in 0.1% Tween-20 (PBS-T) containing 5% non-fat dry milk (A0830; AppliChem, Spain) was used for blocking. Serum/plasma was titrated in two steps in a PBS-T solution containing 3% non-fat dry milk. Anti-mouse IgG horseradish peroxidase-linked secondary antibodies (NXA931; GE Healthcare, USA) were used to detect mouse IgG. Serum of a rabbit immunized with marmoset IgG and anti-rabbit IgG horseradish peroxidase-linked secondary antibodies (NA934V; GE Healthcare, USA) were used to detect marmoset IgG. A Tetramethylbenzidine solution (NIIOPiK, Russia) was used as a chromogenic agent. The reaction was stopped by adding 1 M H_2_SO_4_; optical density was measured at 450 nm (OD_450_) using a Multiskan FC microplate reader (Thermo Fisher Scientific, USA). The IgG titer was defined as the maximum serum dilution at which the OD_450_ value of the serum sample from the immunized animal exceeded that of the control serum/plasma (serum/plasma of the control animal or animal before immunization) more than twofold.

**Determination of the titer of neutralizing antibodies**

The titer of virus-neutralizing antibodies (VNAs) in the plasma of immunized animals was determined in a neutralization reaction (NR) by suppressing the cytopathic effect caused by the MERS-CoV virus (MERS-CoV EMC/2012) in the monolayer of Vero B cells. The neutralization reaction was carried out in the “constant viral dose/serum dilution” mode. Monkey plasma was incubated at 56°C for 30 min to remove non-specific inhibitors. All serum samples were diluted in a DMEM medium supplemented with 2% inactivated fetal bovine serum, starting from the 1 : 10 ratio, then with two-fold dilution to 1 : 5,120. Dilutions of the MERS-CoV virus suspension were prepared in a DMEM medium supplemented with 2% inactivated fetal bovine serum. The concentration of the MERS-CoV virus in the prepared dilution was 1,000 TCID50/ml. A mixture of equal volumes of plasma and the virus suspension was incubated at 37°C for 60 min. Vero B cells were plated in 96-well plates at 4 × 10^4^ cells per well at a volume of 100 μl and then supplemented with 100 μl of the mixture of plasma and the virus suspension. The cytopathic effect was assessed after four days. The VNA titer of the studied plasma was defined as its highest dilution at which the cytopathic effect was suppressed in two out of three wells (compared to the control serum samples).

**Analysis of the T cell response (lymphocyte proliferation assay) and production of interferon gamma (IFN-gamma) in mice**

The mice were euthanized on day eight after immunization. The spleens were collected and homogenized through a 100-μm sieve in sterile PBS. Splenocytes were isolated by Ficoll (1.09 g/ml; PanEco, Russia) density gradient centrifugation (800 *g*, 30 min). For T cell proliferation assay, the splenocytes were stained with carboxyfluorescein using the Carboxyfluorescein succinimidyl ester (CFSE) tracer kit (Invitrogen, USA) as previously described [17]. Cells were seeded in 96-well plates at 2 × 10^5^ cells per well in a RPMI 1640 medium and re-stimulated with the recombinant MERS-CoV S protein (40069-V08B; Sino Biological) at 1 μg per well. After 72 h, the media were collected for a IFN-gamma analysis and the cells were harvested, washed with PBS, stained with antibodies specific to CD3, CD4, and CD8: allophycocyanin (APC)-labelled anti-CD3, APC– Cy7-labelled anti-CD8, and phycoerythrin-labelled anti-CD4 (BD Biosciences, USA), and then fixed in 1% paraformaldehyde. Proliferating CD4^+^ and CD8^+^ T lymphocytes were evaluated in the cell mixture using a BD FACS Aria III flow cytometer (BD Biosciences). The resulting percentage of proliferating cells (X) was determined using the following formula: X = %st – %, where %st is the percentage of proliferating cells after splenocyte re-stimulation with recombinant MERS-CoV S glycoprotein, and % is the percentage of proliferating cells in the absence of splenocyte re-stimulation (intact cells).

The concentration of IFN-gamma in the medium was measured by ELISA using a commercial kit (mouse IFN-γ ELISA kit; Invitrogen) according to the manufacturer’s protocol. The increase in the concentration of IFN-gamma was determined using the following formula: X = Cst/Cint, where X is the fold increase in the concentration of IFN-gamma, Cst is the concentration of IFN-gamma in the medium of stimulated cells (pg/ ml), and Cint is the concentration of IFN-gamma in the medium of unstimulated (intact) cells (pg/ml).

**Assessment of the protective efficacy**

The protective efficacy of the vaccine was studied in a model of lethal infection in transgenic mice carrying the human DPP4 receptor gene and obtained by crossing homozygous transgenic hDPP4+/+ males and non-transgenic C57BL/6 females. The animals were immunized intramuscularly twice successively with component 1 and then component 2 with a 21-day interval. Seven days after the injection of component 2, the mice were infected intranasally with the MERS-CoV virus (MERS-CoV EMC/2012) at a dose of 100 LD_50_ per animal, and then the survival rate was analyzed for a period of 30 days.

**Preclinical safety study**

Preclinical studies of the safety of the combined vector vaccine against MERS were conducted in collaboration with the Autonomous Non-commercial Organization “Institute of Biomedical Research and Technology” and FSAEI HE I.M. Sechenov First Moscow State Medical University, in compliance with the Guidelines for Preclinical Trials of Medicinal Products [18] and Guidelines for experimental (preclinical) study of new pharmacological substances [19]. The safety study included the analysis of the toxicity of a single and repeated administration, as well as sn assessment of the reproductive and ontogenetic toxicity, immunogenicity, and allergenicity. A total of 670 mice, 725 rats, 24 rabbits, 120 guinea pigs, and six common marmosets were used in the preclinical safety study.

Tolerability of the vaccine in primates was analyzed daily by assessing the physical condition of the animals and based on the presence of general symptoms of intoxication, which included an assessment of behavior, appearance, and physiological functions. Vaccine tolerance in marmosets was studied in the laboratory by monitoring the body weight, rectal temperature, and blood biochemical parameters: total bilirubin, direct bilirubin, aspartate aminotransferase (AST), alanine aminotransferase (ALT), creatinine, total protein, and alkaline phosphatase (ALP). The studies were carried out on fully automatic analyzers CA-180 and B-200 (Furuno, Japan) using DiaSys reagent kits (Germany).

**Statistical analysis**

Statistical analysis of the data was performed using the GraphPad 7.0 software. Either the Student’s t-test for independent samples or the Mann–Whitney U-test was used for the analysis of the data of unpaired samples depending on the data distribution normality [20]. Either the Student’s t-test for paired samples or Wilcoxon’s test was used for the analysis of the data of related samples depending on the data distribution normality [20]. Distribution normality was determined using the generalized D’Agostino–Pearson test [21].

## RESULTS

**Immunization of the animals with the combined vector vaccine induces a robust long-term humoral immune response to MERS-CoV glycoprotein in mice and primates**

**Fig. 1:**
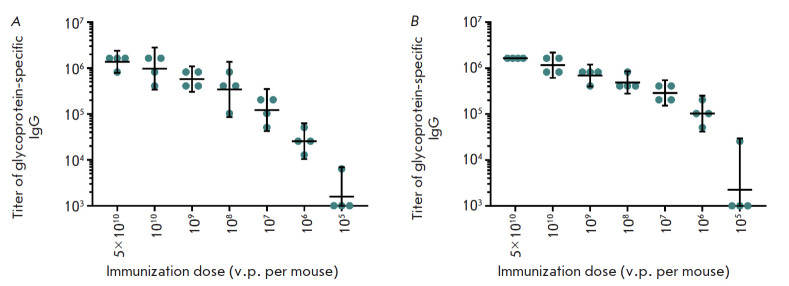
Titers of glycoprotein-specific IgGs in the serum of immunized animals two weeks (*A*) and four weeks (*B*) after boosting of the vaccination. The abscissa axis represents immunization doses (v.p. per mouse); the ordinate axis shows reciprocal IgG titers. The geometric mean titers and 95% confidence intervals are indicated

In order to select an effective dose, mice were immunized intramuscularly with the vaccine at doses of 105–1010, 5 × 10^10^ v.p. per mouse; serum samples were collected, and the titers of glycoprotein-specific antibodies were analyzed two and four weeks after immunization. Next, the intensity of the post-vaccination humoral immune response was assessed based on the titer of glycoprotein-specific IgG (*Fig. 1*).

**Fig. 2:**
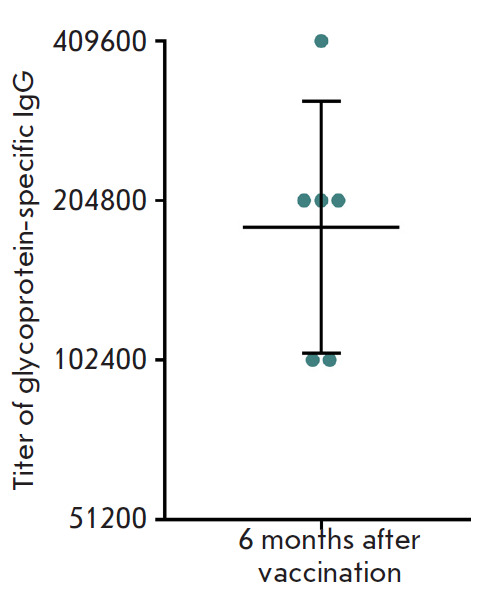
Titers of glycoprotein-specific IgGs in the serum of immunized animals six months after vaccination. The ordinate axis shows reciprocal IgG titers. The geometric mean titer and the 95% confidence interval (n = 6) are indicated

Analysis of the obtained results demonstrates a dose-dependent increase in the serum titer of glycoprotein-specific IgG. The minimum dose of the combined vector vaccine required to induce a robust humoral immune response was 106 v.p. per mouse for all vaccinated animals. Analysis of the duration of post-vaccination humoral immunity showed that glycoprotein-specific antibodies were detected at a high titer in the mouse serum six months after immunization (the geometric mean titer was 1 : 182,456, *Fig. 2*).

**Fig. 3:**
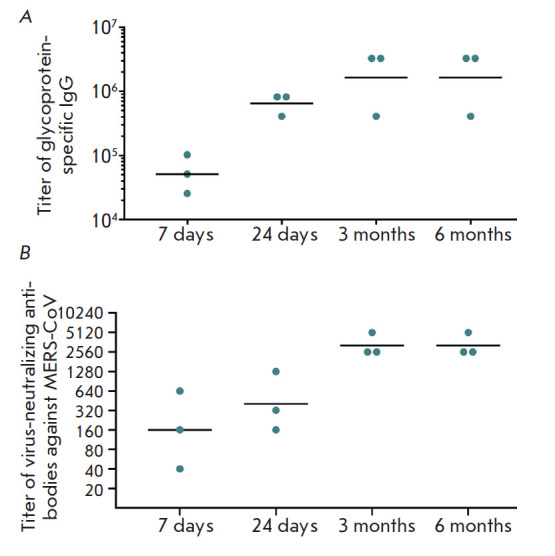
*A *– Titers of glycoprotein-specific IgG in the plasma of immunized marmosets after vaccination. IgG titers are shown on the ordinate axis; time after immunization is represented on the abscissa axis. Individual titers for each studied animal and the geometric mean titer (n = 3) are indicated. *B *– Titers of virus-neutralizing antibodies in the plasma of immunized marmosets after vaccination. Virus-neutralizing antibody titers are shown on the ordinate axis; time after immunization is represented on the abscissa axis. Individual titers for each studied animal and the geometric mean titer (n = 3) are indicated

Next, we studied the level of humoral immunity in primates vaccinated with the developed vaccine. In order to determine the level of humoral immunity in common marmosets (*C. jacchus*), they were immunized with the combined vaccine according to the regimen intended for clinical use, i.e. successively with component 1 (at a dose of 1011 v.p. per animal) and then component 2 (at a dose of 1011 v.p. per animal) with a 21-day interval. Further, plasma samples were collected from animals for the analysis of the titer of glycoprotein-specific IgG seven and 24 days, as well as three and six months, after the boosting of immunization (*Fig. 3A*). Immunization of primates was shown to induce robust humoral immunity, which persists for at least six months. For instance, the titers of glycoprotein-specific IgG in primates six months after immunization did not differ from the titers after three months, which is an indication of the induction of long-term immunity. Analysis of the titer of neutralizing antibodies to the MERS-CoV virus in the plasma of immunized monkeys showed that VNAs were detected in the animals as early as seven days after booster immunization, while the maximum VNA titer was reached three and six months after immunization (*Fig. 3B*). No VNAs were detected in the plasma of the control animals and the animals before immunization.

Thus, our analysis of the level of post-vaccination immunity showed that immunization of mice and primates induces a robust humoral immune response, which persists for at least six months after immunization.

**Immunization of mice with the candidate vaccine induces a robust cellular immune response**

**Fig. 4:**
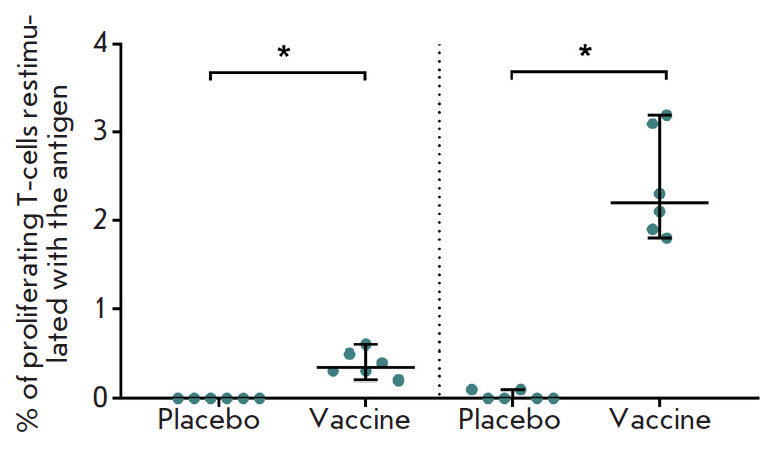
Study of the lymphoproliferative activity of splenocytes in mice immunized with the vaccine or placebo. The levels (in %) of proliferating CD4^+^ and CD8^+^ T cells re-stimulated with recombinant MERS-CoV S glycoprotein on the 18th day after vaccination are presented. Medians of the percentage of proliferating cells after re-stimulation and 95% CI for the median for each group (n = 6) are indicated. * – *p * < 0.05

In order to assess the level of post-vaccination cellular immunity, the mice were immunized with the candidate vaccine against MERS once at a dose of 107 v.p. per mouse. Spleens were collected from the animals 18 days after immunization; splenocytes were isolated, and the number of proliferating CD4^+^ and CD8^+^ T lymphocytes was determined in the splenocyte culture *in vitro *after cell re-stimulation with the recombinant MERS-CoV S protein (*Fig. 4*). The obtained data demonstrate that introduction of the combined vector vaccine induces the formation of S-specific CD4^+^ and CD8^+^ T cells.

**Fig. 5:**
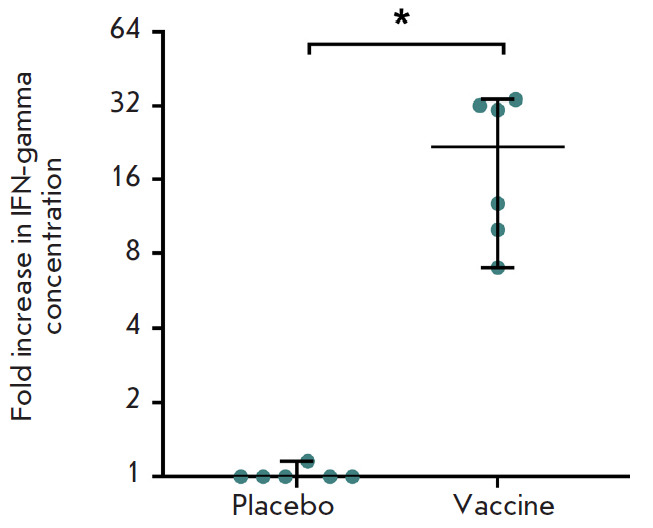
Increase in the concentration of IFN-gamma in the splenocyte media of immunized and non-immunized mice after re-stimulation with recombinant MERS-CoV S glycoprotein. Median increase in the concentration of IFN-gamma after re-stimulation and 95% CI for the median for each group (n = 6) are indicated. * – *p* < 0.05

Activation of cellular immunity was also analyzed by measuring the expression of IFN-gamma. The results of the study of an increase in the IFN-gamma concentration in an *in vitro *culture of mouse splenocytes after repeated stimulation of the cells with the recombinant MERS-CoV S protein are presented in *Fig. 5*. Administration of the vaccine increased the concentration of IFN-gamma in the medium upon stimulation of the splenocytes of immunized mice with the MERS-CoV S glycoprotein. The concentration of IFN-gamma in the medium increased by an average of 22 times.

Summarizing the data of our analysis of the antigen-specific lymphoproliferative activity of CD4^+^ and CD8^+^ T cells and the level of IFN-gamma expression by re-stimulated splenocytes, we can conclude that immunization of animals with the combined vaccine against MERS results in the formation of glycoprotein-specific cellular immunity.

**Combined vector vaccine protects animals against lethal infection with the MERS-CoV virus**

**Fig. 6:**
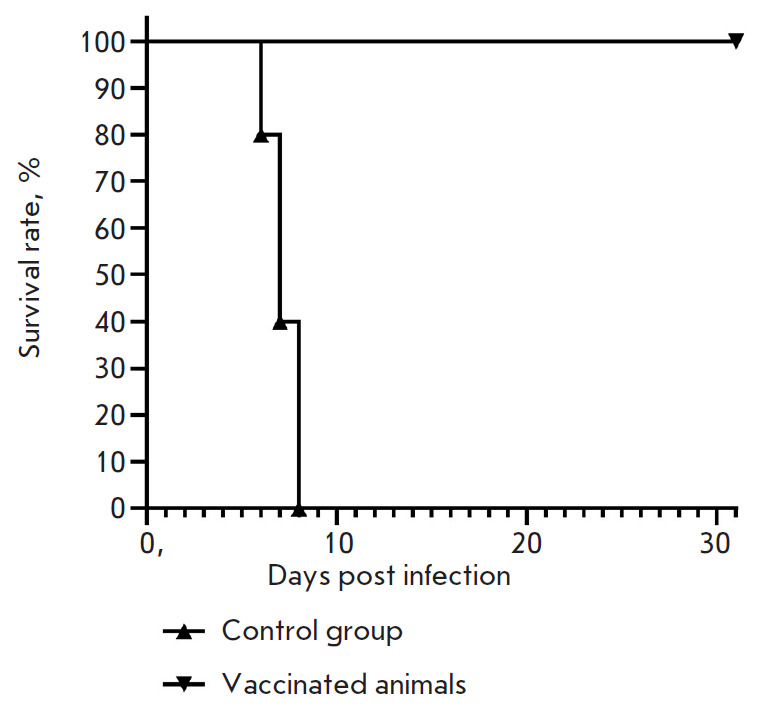
Survival of vaccinated (n = 10) and non-vaccinated (control group, n = 10) animals after a lethal infection of MERS-CoV. The ordinate axis shows the survival rate of animals (%). The abscissa axis represents time after immunization (days)

The study was carried out in a model of lethal infection caused by MERS-CoV in transgenic mice carrying the human DPP4 receptor gene. Mice were immunized successively with component 1 and then component 2 with a 21-day interval. One week after administration of component 2 of the vaccine, animals were infected intranasally with the MERS-CoV virus (MERS-CoV EMC/2012) at a dose of 100 LD_50_ per animal, and the survival rate was assessed during 30 days. Immunization of the animals with the combined vector vaccine was shown to protect 100% of animals from lethal infection caused by the MERS-CoV virus. All control (unvaccinated) animals died (*Fig. 6*).

**The combined vector vaccine for the prevention of MERS has favorable safety and tolerability profiles in animals**

General and specific toxicity (the toxicity of single and repeated administration, assessment of the local irritation effect, immunotoxicity, allergenic properties, and reproductive toxicity) were evaluated in rodents (mice, rats, and guinea pigs) and large animals (rabbits). The combined vector vaccine against MERS did not cause any toxic effects, did not have an allergenic or immunotoxic effect, did not affect the generative function, did not have a local irritation effect, and can be recommended for clinical studies.

**Fig. 7:**
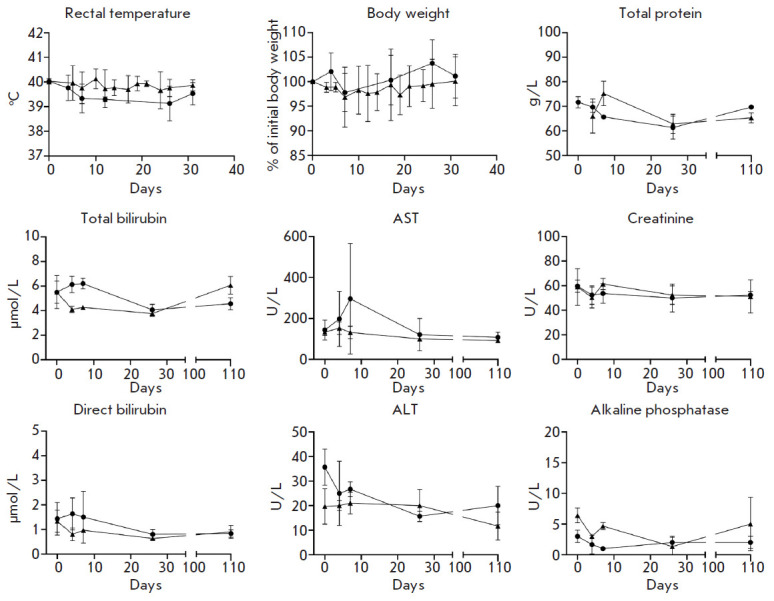
Rectal temperature, body weight, and biochemical blood parameters in primates (common marmosets) immunized with the combined vector vaccine against MERS (indicated by triangles) and control animals (marked by circles)

Vaccine tolerability was also studied in primates. No abnormalities in the analyzed parameters of physical condition (behavioral reactions, appearance, and physiological functions) were found in the animals immunized with the combined vector vaccine against MERS and the control animals during the observation period. Rectal temperature, changes in body weight, and biochemical parameters were within the normal range for the species in all animals during the experiment (*Fig. 7*). Summarizing the obtained data, we can conclude that the combined vector vaccine against MERS has shown good tolerability in the common marmoset model.

## DISCUSSION

Currently, there are no specific prophylactic and therapeutic agents against the Middle East Respiratory Syndrome in the World. Intensive studies on the development of vaccines for this disease are currently underway in the United States, Germany, Korea, China, Great Britain, and other countries. Among the prophylactic drugs with the highest efficiency demonstrated in preclinical studies, the following candidate vaccines can be mentioned: vaccines based on adenoviral vectors (Ad5, Ad41, ChAdOx1) [22, 23], Modified Vaccinia virus Ankara (MVA) [24] encoding MERS-CoV protective antigen S, as well as preparations of recombinant MERS-CoV protective antigen S [25, 26]. Two drugs are currently undergoing clinical trials: two vaccines based on recombinant viral vectors MERS001 (based on chimpanzee adenovirus, phase 1) [27] and MVA-MERS-S (based on vaccinia virus, phase 2) [28]. Clinical studies of the first phase of a vaccine based on plasmid DNA (GLS-5300), as well as a vaccine based on a vaccinia virus (MVA-MERS-S), have been completed [29, 30].

All vaccines that have reached clinical trials are based on MERS-CoV S glycoprotein. This glycoprotein performs one of the most important roles in the viral life cycle: it enables virus internalization via interaction with the DPP4 receptor on the cell surface. Neutralization of this interaction limits penetration of the virus into the cell, thus decreasing its replication.

Since the formation of not only a humoral, but also cellular immune response is important for protection against MERS, the development of vaccines based on recombinant viral vectors seems promising. Such vectors effectively deliver antigen-encoding genetic material to the cells, which results in the cellular expression of the antigen and induction of a robust cellular and humoral immunity. An important property of recombinant viral vectors is that they induce protective immunity as early as after the first or second immunization, which is extremely important when developing a vaccine for the prevention of dangerous and extremely dangerous infections and is intended for use during an epidemic or in the case of an infection that spreads beyond non-endemic areas.

We have conducted a study of the immunogenicity of various forms of MERS-CoV S glycoprotein: full-length glycoprotein (S), full-length glycoprotein with the transmembrane domain of the G protein (S-G) of the vesicular stomatitis virus, a secreted glycoprotein receptor-binding domain (RBD), a secreted glycoprotein RBD fused to the Fc fragment of human IgG1 (RBD-Fc), and the membrane form of the glycoprotein RBD (RBD-G) [31]. The obtained data demonstrated that the membrane form of the RBD is the most effective in inducing a robust cellular immune response, while full-length glycoprotein is most efficient in inducing a robust cellular immunity. When choosing an immunization regimen, one should take into account the fact that repeated heterologous vaccination, which involves the use of two different recombinant viral vectors for primary and secondary immunization, is advisable for inducing long-term immunity. For this reason, the combined vaccine against MERS included two recombinant vectors based on human adenovirus serotypes 26 and 5. Component 1 included the rAd26- RBD-G recombinant vector, while component 2 was comprised of two recombinant vectors: rAd5-S and rAd5-RBD-G.

Studies of the immunogenicity of the combined vector vaccine revealed the induction of long-term humoral immunity in mice, while the mean titer of glycoprotein-specific antibodies equaled 1 : 121,775 two weeks after vaccination at a dose of 107 v.p. per mouse. A similar antibody titer was observed by Alharbi et al. in mice 28 days after immunization with a vaccine against MERS based on chimpanzee adenovirus ChAdOx1 MERS [12]; however, the authors used a dose of 108 v.p. per mouse for immunization. In another study by Munster et al. [13], immunization of transgenic mice carrying the human DPP4 receptor gene with a ChAdOx1 MERS vaccine at a dose 108 v.p. per mouse was shown to protect 100% of the animals from a lethal infection with MERS-CoV. Hashem et al. developed a rAd5-based drug carrying the MERS-CoV S1 sequence and demonstrated that repeated immunization of mice with the drug at a dose of 109 v.p. per mouse induced a humoral immune response [32]. The titer of glycoprotein-specific IgG was 1 : 70,000 three weeks after the second immunization (one and a half months from the beginning of immunization); the drug also provided 100% protection to animals from MERS-CoV infection [32].

Glycoprotein-specific antibodies were found at a titer range of 1 : 25,600 to 1 : 102,400 in the plasma of the animals as early as a week after the boosting of immunization. It is important to note that Muthumani et al. [33] detected glycoprotein-specific antibodies at a titer of 1 : 20,000 for a period of six weeks in primates after long-term thrice immunization with a DNA vaccine; the authors also showed that immunization of primates with the DNA vaccine protects them from a MERS-CoV infection.

The study of post-vaccination humoral immunity in mice and primates demonstrated that an intense humoral immune response persists in the animals for at least six months after vaccination. Analysis of the cellular component of the immunity in the mice showed that administration of the developed vaccine induces a robust cellular response. It is important to note that not only a CD4^+^ but also CD8^+^ T cell response is observed, which can play an important role in protection against MERS-CoV [34, 35]

Having completed studies of the immunogenicity of the combined vector vaccine in a model of lethal infection in transgenic mice carrying the human DPP4 receptor gene, we studied the protective effect of the vaccine. The vaccine was shown to provide 100% protection to animals from lethal infections of MERS-CoV. Our series of preclinical studies of vaccine safety revealed no contraindications for the clinical testing of the developed vaccine.

## CONCLUSION

In this work, we have studied the immunogenicity and safety of a combined vector vaccine for the prevention of the Middle East Respiratory Syndrome. The following conclusions were obtained:

Vaccination of animals with the vaccine induces a robust humoral immune response to the MERS-CoV S glycoprotein persisting for at least six months.

Vaccination of animals induces a robust cellular immune response to the MERS-CoV S glycoprotein.

Vaccination of animals induces a protective immune response, which protects 100% of animals from a lethal infection of MERS-CoV.

Preclinical studies of the vaccine safety did not reveal any contraindications to clinical testing.

## Abbreviations

95% CI: 95% confidence interval
Ad5: recombinant human serotype 5 adenovirus
Ad26: recombinant human serotype 26 adenovirus
Ad41: recombinant human serotype 41 adenovirus
APC: allophycocyanin
ChAdOx1: recombinant chimpanzee adenovirus vector
DPP4: dipeptidyl peptidase 4
MVA: modified vaccinia virus Ankara
RBD: receptor-binding domain of MERS-CoV S glycoprotein
RBD-Fc: receptor-binding domain of MERS-CoV S glycoprotein fused to the Fc domain of human IgG1
RBD-G: receptor-binding domain of MERS-CoV S glycoprotein fused to the transmembrane domain of the glycoprotein G of vesicular stomatitis virus
S: MERS-CoV glycoprotein
S-G: MERS-CoV glycoprotein with the transmembrane domain of the glycoprotein G of vesicular stomatitis virus
ALT: alanine aminotransferase
AST: aspartate aminotransferase
MERS: Middle East Respiratory Syndrome
MERS-CoV: Middle East Respiratory Syndrome coronavirus
v.p.: viral particles
IFN-gamma: interferon gamma
ALP: alkaline phosphatase
